# Correction to: Arthroscopic treatment of focal osteochondral lesions of the first metatarsophalangeal joint

**DOI:** 10.1186/s13018-019-1456-7

**Published:** 2019-12-23

**Authors:** Ersin Kuyucu, Harun Mutlu, Serhat Mutlu, Baris Gülenç, Mehmet Erdil

**Affiliations:** 1 Orthopedics and Traumatology, Istanbul Medipol University, Istanbul, Turkey; 2 Orthopedics and Traumatology, Taksim Ilkyardım Training and Education Hospital, Istanbul, Turkey; 3 Orthopedics and Traumatology, Kanuni Sultan Süleyman Training and Education Hospital, Istanbul, Turkey; 4 TEM Avrupa Otoyolu Göztepe Çıkışı No:1, Bağcilar, Istanbul, Turkey

**Correction to: J Orthopedic Surg Res**

**https://doi.org/10.1186/s13018-017-0562-7**

This article [2] was published twice [1] due to a production error. The original article [1] should be considered the version of record and used for citation purposes. The publisher apologizes to the authors and readers for the error and any inconvenience caused.

## References

[1] Kuyucu (2017). Arthroscopic treatment of focal osteochondral lesions of the first metatarsophalangeal joint. J Orthopaedic Surg Res.

[2] Kuyucu (2017). Arthroscopic treatment of focal osteochondral lesions of the first metatarsophalangeal joint. J Orthopaedic Surg Res.

